# An Amygdala‐hippocampus Circuit for Endocannabinoid Modulation of Anxiety Avoidance

**DOI:** 10.1002/advs.202505121

**Published:** 2025-06-16

**Authors:** Bao Xue, Mao‐Xing Zhang, Xiao‐Chen Bi, Shou‐Peng Lai, Xin‐Tian Bie, Yuan Dong, Jian‐Feng Li, Fang Gao, Xia Zhang, Ying Wang

**Affiliations:** ^1^ Neuropsychiatry Research Institute Basic School of Medicine The Affiliated Hospital of Qingdao University Qingdao University Qingdao 266000 China; ^2^ School of Chinese Materia Medica and Yunnan Key Laboratory of Southern Medicinal Utilization Yunnan University of Chinese Medicine Kunming 650500 China; ^3^ Department of Neurobiology School of Basic Medicine Fourth Military Medical University Xi'an 710032 China; ^4^ Departments of Neurology and Psychiatry West China Hospital of Sichuan University Chengdu 610041 China

**Keywords:** 2‐AG synthase, anxiety avoidance, endocannabinoid modulation, neural circuit, opto‐CB1R

## Abstract

Recent studies indicate a therapeutic potential of increased brain endocannabinoids (eCBs) in anxiety disorders, but the underlying brain circuits are still elusive. Here, it is observed that optogenetic inhibition and activation of anterior basolateral amygdala (aBLA) ‐ ventral hippocampus (vHPC) glutamatergic projections respectively decrease and increase anxiety avoidance behaviors. Then, the contributions of eCBs in aBLA–vHPC projections to anxiety avoidance are investigated by employing three newly developed synapse‐ and circuit‐specific eCB‐targeted viral strategies to achieve real‐time monitoring of eCB release, in vivo optogenetic activation of CB1 receptors, and CRISPR‐Cas9 gene knockdown of eCB biosynthesis enzymes. Prominent eCB release are surprisingly found at aBLA–vHPC glutamatergic synapses during anxiety avoidance, suggesting inhibitory effects of increased eCBs in aBLA–vHPC projections on anxiety avoidance. This idea is further supported by findings that specific activation of CB1 receptors at aBLA–vHPC synapses inhibit presynaptic glutamate release and reduce anxiety avoidance. In contrast, specific knockdown of eCB biosynthesis enzymes at aBLA–vHPC synapses reduce eCB levels at aBLA–vHPC glutamatergic synapses and increase anxiety avoidance. Additionally, inhibition of aBLA‐innervated vHPC glutamatergic neurons alleviates anxiety avoidance. Together, these findings reveal counteracting effects of increased eCB signaling in aBLA–vHPC circuits on anxiety avoidance.

## Graphical abstract

This study employs three synapse‐specific endocannabinoids (eCB) probes to monitor dynamic eCB release, activate CB1Rs and knockdown 2‐AG biosynthesis enzymes in glutamatergic anterior basolateral amygdala ‐ ventral hippocampus (aBLA–vHPC) circuit. At aBLA–vHPC circuits, activation of CB1Rs reduce anxiety avoidance, and knockdown of eCB biosynthesis increase anxiety avoidance. The article establishes a base for future clinical interventions of anxiety disorders with precise interference of eCB signaling in aBLA–vHPC circuits.

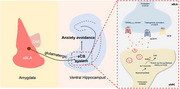

## 1 Introduction

Anxiety disorder is one of the most prevalent mental diseases in the current society under increased life and economy pressure.^[^ ^1^ ^]^ Patients with anxiety disorders often avoid situations that trigger their anxiety, leading to an anxiety‐avoidance cycle.^[^ ^2^ ^]^ Ultimately, continuing to engage in avoidance to prevent feelings of fear or anxiety fuels the cycle and allows both their anxiety and avoidance to grow, producing a negative impact on everyday life. Therefore, reducing unnecessary and extreme avoidance behaviors is important to suspend the development of anxiety disorders.

Endocannabinoids (eCBs) in the brain including anandamide (AEA) and 2‐arachidonoylglycerol (2‐AG), are activity‐dependently produced by postsynaptic neurons and retrogradely bound to presynaptic CB1 receptors (CB1Rs) to suppress presynaptic neurotransmitter release.^[^ ^3^ ^]^ Both preclinical and clinical studies have shown eCB system as a promise therapeutic target in anxiety disorders.^[^ ^4^ ^]^ Genetic knockout or antagonism of CB1Rs can cause increased anxiety‐like behavior.^[^ ^5^ ^]^ One well‐known case is that the anti‐obesity drug rimonabant, a CB1R antagonist, increases the risk of severe anxiety and depression, which leads to market withdrawal in 2008.^[^ ^6^ ^]^ In contrast, synthetic cannabinoids with agonist activity to CB1Rs, or inhibitors of enzymes hydrolyzing AEA or 2‐AG could exert anti‐anxiety effects.^[^ ^7^, ^8^, ^9^ ^]^ However, due to that traditional pharmacology or gene knockout methods are lack of circuit or synapse specificity, the exact neural circuit substrates underlying the effect of eCB signaling changes on anxiety remain unclear, which impedes the development of eCB system‐based medications without off‐target side effects.^[^ ^10^ ^]^

Amygdala is one of the brain regions with abundant CB1Rs and involved in processing emotional responses to stress or stimuli.^[^ ^11^, ^12^ ^]^ Particularly, basolateral amygdala (BLA) and its connections with the medial prefrontal cortex (mPFC) and ventral hippocampus (vHPC) have been reported to modulate anxiety, depression and fear expression.^[^ ^13^, ^14^, ^15^, ^16^, ^17^, ^18^, ^19^ ^]^ We recently reported that an i.p. administration of AEA degradative enzyme inhibitors increased AEA concentrations in the BLA and then produced a cascade events, including activation of BLA astrocyte CB1Rs, elevation of interstitial glutamate concentrations, activation of postsynaptic glutamate receptors, and inhibition of mPFC‐BLA projections, resulting in anxiolytic effects.^[^ ^13^ ^]^ BLA‐vHPC projections have recently been found to mediate acute stress‐elicited anxiety avoidance behaviors,^[^ ^18^, ^19^ ^]^ but whether and how eCB signaling contributes to anxiety avoidance through BLA‐vHPC projections are entirely unknown. Here, we investigated these questions utilizing three projection‐ and synapse‐specific probes. The first probe is the eCB biosensor to monitor the real‐time dynamic eCB release in specific circuits and synapses.^[^ ^20^ ^]^ The second probe is the recently developed opto‐CB1R (opCB1R) strategy that uses a light‐sensitive CB1R chimera, resulting in a precise activation of opCB1Rs in the circuit‐ and synapse‐specific manner.^[^ ^21^ ^]^ The third probe is using *Staphylococcus aureus* Cas9 (SaCas9) gene editing to knockout the eCB 2‐AG biosynthase diacylglycerol lipase α (DAGLα) in a circuit‐ and synapse‐specific way.^[^ ^22^, ^23^ ^]^ We found counteracting effects of naturally and artificially increased eCB signaling in anterior BLA (aBLA)‐vHPC circuits on anxiety avoidance induced by acute anxiety stress.

## 2 Results

### 2.1 Increased Neuronal Activity in aBLA–vHPC Circuits During Anxiety Avoidance

**Figure 1:**
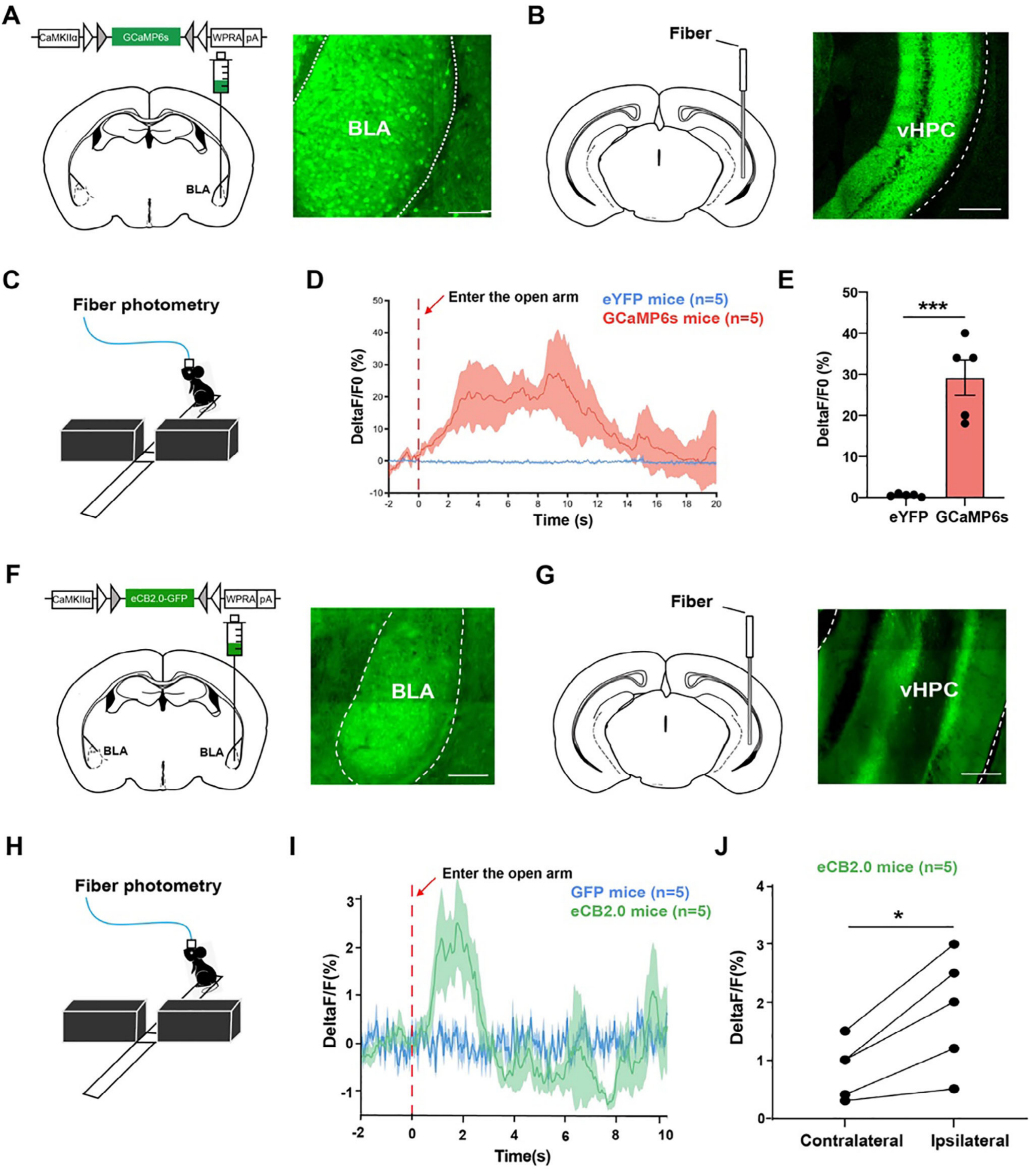
Increased neuronal activity and endocannabinoids (eCB) release in anterior basolateral amygdala ventral hippocampus (aBLA–vHPC) circuits during anxiety avoidance. A) Left: Schematic diagram of CaMKIIα‐GCaMP6s virus injections into the aBLA. Right: Representative image showing expression of CaMKIIα‐GCaMP6s virus in aBLA, scale bar = 100 µm. B) Left: Schematic diagram of recording optic fiber implantation into the ventral hippocampus (vHPC). Right: Representative image showing expression of CaMKIIα‐ GCaMP6s virus in aBLA to vHPC terminals, scale bar = 200 µm. C) Diagram of fiber photometry recording in elevated plus maze (EPM) test. D) Averaged trace for calcium signals in GCaMP6s mice (red line) and eYFP mice (blue line). The vertical dashed line indicates the mice entering the open arm. E) Quantification of open arm stress‐induced activation peak deltaF/F (%) in GCaMP6s mice (*n* = 5) and eYFP mice (*n* = 5). Independent *t*‐test, *t* = 6.630, df = 8, *p* = 0.0002, ^***^ *p* < 0.0001. F) Left: Schematic diagram of CaMKIIα‐eCB2.0 virus injections into the aBLA. Right: Representative image showing expression of CaMKIIα‐eCB2.0 virus in aBLA, scale bar = 100 µm. G) Left: Schematic diagram of recording optic fiber implantation into the vHPC. Right: Representative image showing expression of CaMKIIα‐ eCB2.0 virus in aBLA to vHPC terminals, scale bar = 200 µm. H) Diagram of fiber photometry recording in EPM test. I) Averaged trace for eCB release signals in eCB2.0 mice (green line) and GFP mice (blue line). J) Quantification of ipsilateral/contralateral open arm stress‐induced activation peak deltaF/F (%) in eCB2.0 mice. Paired *t*‐test, *t* = 4.117, df = 4, *p* = 0.0146, ^*^ *p* < 0.05. Data are shown as the mean ± SEM.

It has been reported that aBLA and vHPC are structurally connected and functionally coupled in anxiety.^[^ ^18^, ^19^ ^]^ First, we examined possible changes of neuronal activity in the aBLA–vHPC circuit in response to anxiety stress. We used in vivo fiber photometry to monitor real‐time neuronal activity changes of aBLA glutamatergic neurons during the elevated plus maze (EPM) where mice feel stressful in open arms (Figure S1A, Supporting Information). The genetically encoded Ca^2+^ indicator CaMKIIα‐GCaMP6s was delivered into the aBLA of wild type mice. Three weeks later, an optic fiber was implanted above the virus injection site (Figure S1B,C, Supporting Information). After 1 week recovery, fluorescence recording during the EPM task was performed. The calcium activity of aBLA glutamatergic neurons transiently increased when mice were entering open arms (Figure S1D,E, Supporting Information), indicating that aBLA glutamatergic neurons are activated by anxiety‐like stress. With GCaMP6s virus expression, we also found that aBLA glutamatergic neurons sent strong GCaMP6s‐positive projections to well‐known emotion‐related areas, including the mPFC, nucleus accumbens, bed nucleus of the stria terminalis, and vHPC (Figure S2, Supporting Information). Because recent studies showed that the aBLA–vHPC circuit bidirectionally modulated anxiety avoidance behaviors, we further used fiber photometry to monitor the activity of aBLA glutamatergic projections in the vHPC during the EPM test with GCaMP6s delivered into the aBLA and optic fiber implanted in the vHPC (**Figure** **1**A–C). The calcium activity of aBLA–vHPC glutamatergic projections also started to increase when mice were entering open arms but showed a prolonged increase than that of aBLA glutamatergic neurons (Figure 1D,E). Our GCaMP6s results suggest that when mice were entering the stressful open arms in the EPM, aBLA–vHPC glutamatergic projections were activated to increase the release of glutamate and subsequently led to anxiety avoidance.

### 2.2 Increased eCB Release in aBLA–vHPC Circuits During Anxiety Avoidance

Given that brain eCBs are activity dependently produced by postsynaptic neurons,^[^ ^3^ ^]^ activation of aBLA‐vHPC glutamatergic projections during anxiety avoidance indicates that the same stressful stimuli may increase eCB release in the aBLA–vHPC synapses. We therefore investigated this hypothesis with an eCB biosensor (GRABeCB2.0).^[^ ^20^ ^]^ We observed a robust fluorescence response to eCB concentration changes (Figure 1F). The eCB biosensor was delivered into the left aBLA and an optic fiber was implanted in the left vHPC (Figure 1F,G), followed by fiber photometry monitor of the eCB release during anxiety avoidance with the EPM (Figure 1H). We observed that open arm entering‐induced anxiety stress significantly increased the release of eCBs in the aBLA–vHPC circuit (Figure 1I,J). Interestingly, the eCB release in the aBLA–vHPC circuit has direction bias, where more eCBs were released when mice were entering the open arm ipsilateral to the optic fiber than contralateral side (Figure 1J). These results indicate that open arm entering‐induced anxiety stress significantly activated postsynaptic vHPC neurons to increase the release of eCBs in aBLA–vHPC synapses.

### 2.3 aBLA Glutamatergic Projections to vHPC Bidirectionally Modulate Anxiety Avoidance

**Figure 2:**
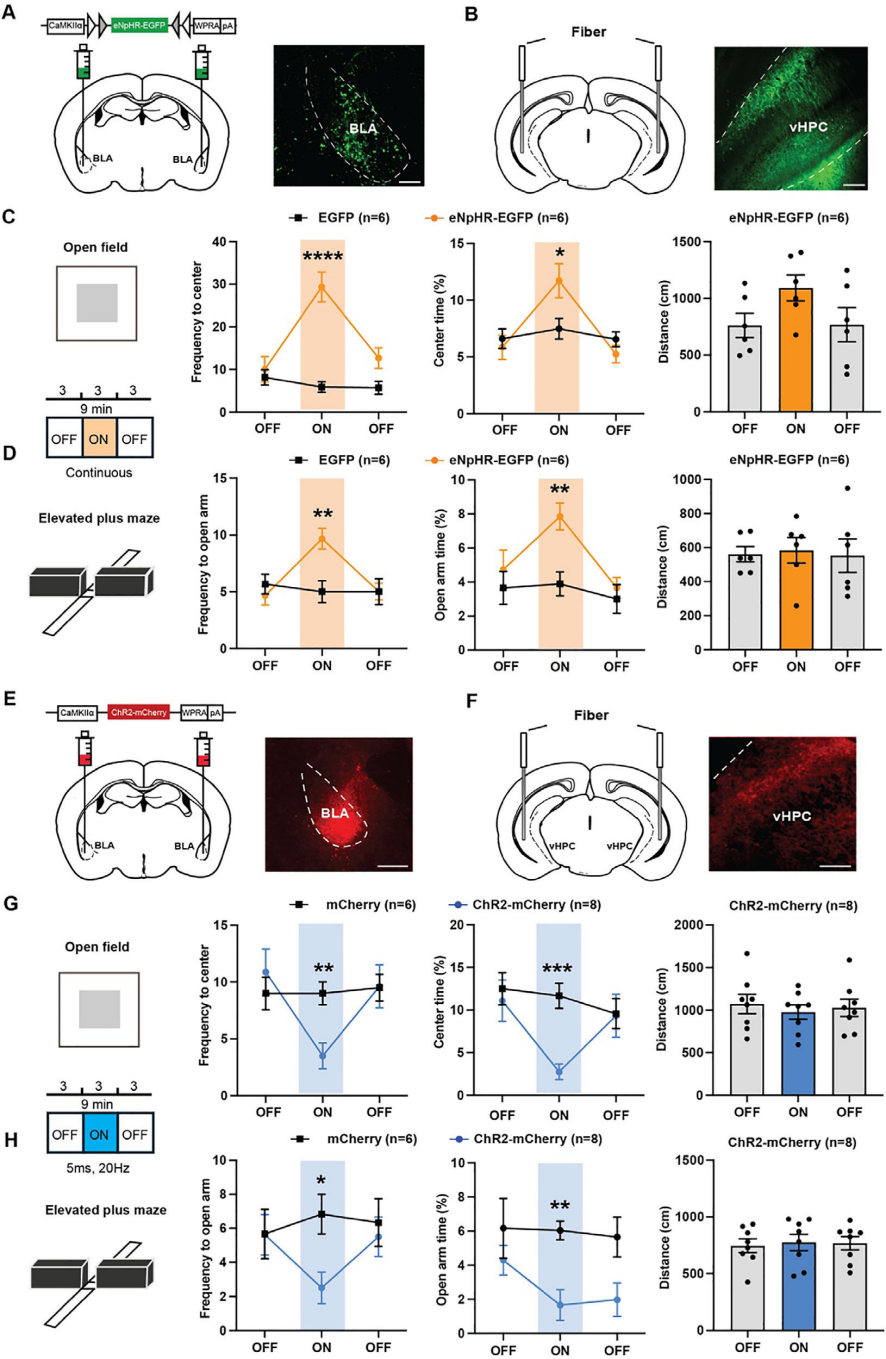
Anterior basolateral amygdala (aBLA) glutamatergic projections to ventral hippocampus (vHPC) bidirectionally modulate anxiety avoidance. A,E) Left: Schematic diagram of CaMKIIα‐eNpHR/ChR2 virus injections into the aBLA. Right: Representative image showing expression of CaMKIIα‐eNpHR/ChR2 virus in aBLA, scale bar = 100 µm in A and 200 µm in E. B,F) Left: Schematic diagram of optic fiber implantation into the vHPC. Right: Representative image showing expression of CaMKIIα‐eNpHR virus in aBLA to vHPC terminals, scale bar = 100 µm. C) Quantification of frequency to center zone (2 × 3 mixed two‐way ANOVA, significant interaction between group and light on, F(2, 20) = 12.74, *p* = 0.0003, ^***^ *p* < 0.001; independent *t*‐test, ON: *t* = 6.279, df = 10, *p* < 0.0001, ^****^ *p* < 0.0001), percentage time spent in the center zone of open field test (OFT) (2 × 3 mixed two‐way ANOVA, significant interaction between group and light on, F(2, 20) = 4.879, *p* = 0.0188, ^*^ *p* < 0.05; independent *t*‐test, ON: *t* = 2.436, df = 10, *p* = 0.0351, ^*^ *p* < 0.05) and total locomotor distance for eNpHR group. D) Quantification of frequency to open arm (2 × 3 mixed two‐way ANOVA, significant interaction between group and light on, F(2,20) = 5.346, *p* = 0.0103, ^*^ *p* < 0.05; independent *t*‐test, ON: *t* = 3.500, df = 10, *p* = 0.0057, ^**^ *p* < 0.01), percentage time spent in the open arm of elevated plus maze (EPM) (2 × 3 mixed two‐way ANOVA, no significant interaction between group and light on, F(2,20) = 2.265, *p* = 0.1299; independent *t*‐test, ON: *t* = 3.745, df = 10, *p* = 0.0038, ^**^ *p* < 0.01) and total locomotor distance for eNpHR group. G) Quantification of frequency to center zone (2 × 3 mixed two‐way ANOVA, significant interaction between group and light on, F(2, 24) = 5.303, *p* = 0.0124, ^*^ *p* < 0.05; independent *t*‐test, ON: *t* = 3.459, df = 12, *p* = 0.0047, ^**^ *p* < 0.01), percentage time spent in the center zone of OFT (2 × 3 mixed two‐way ANOVA, significant interaction between group and light on, F(2, 24) = 3.873, *p* = 0.0349, ^*^ *p* < 0.05; independent *t*‐test, ON: *t* = 5.370, df = 12, *p* = 0.0002, ^***^ *p* < 0.001) and total locomotor distance for ChR2 group. H) Quantification of frequency to open arm (2 × 3 mixed two‐way ANOVA, significant interaction between group and light on, F(2,24) = 3.643, *p* = 0.0415, ^*^ *p* < 0.05; independent *t*‐test, ON: *t* = 2.949, df = 12, *p* = 0.0122, ^*^ *p* < 0.05), percentage time spent in the open arm of EPM (2 × 3 mixed two‐way ANOVA group, no significant interaction between group and light on, F(2,24) = 0.8583, *p* = 0.4365; independent *t*‐test, ON: *t* = 3.795, df = 12, *p* = 0.0026, ^**^ *p* < 0.01) and total locomotor distance for ChR2 group. Data are shown as the mean ± SEM.

To verify the role of aBLA–vHPC glutamatergic circuit in anxiety avoidance behaviors, we injected AAV2/9‐CaMKIIα‐eNpHR3.0‐EGFP or AAV2/9‐CaMKIIα‐hChR2‐mCherry into aBLAs. Three weeks later, we implanted the optic fiber in vHPCs to bidirectionally modulate the activity of aBLA–vHPC glutamatergic terminals (**Figure** **2**A,B,E,F). After 1 week recovery, approach and/or avoidance behaviors were recorded in the open field test (OFT) and EPM. The function of ChR2 and eNpHR3.0 to activate or inhibit neuronal activities was validated by in vitro electrophysiological test (Figure S3, Supporting Information). To allow for within‐subject and within‐session comparisons in addition to group comparisons, we tested mice on a single 9 min session on both OFT and EPM with three 3‐min epochs, beginning with a light‐off (OFF) baseline epoch, followed by a light‐on (ON) epoch with direct current yellow (596 nm) light or 20 Hz blue (473 nm) light pulses, then alternating back to a second OFF epoch. During the light‐on epoch in the OFT, we observed that compared with EGFP group, the number of times into the center and the time spent in the center area (%) in the eNpHR3.0 group were significantly increased (Figure 2C). During the light‐on epoch in the EPM, the eNpHR3.0 mice explored open arms more often than EGFP control mice, and their time spent in open arms was significantly increased (Figure 2D). These data demonstrate that inhibition of aBLA–vHPC glutamatergic circuits is sufficient to inhibit anxiety avoidance behaviors. As expected, during the light‐on epoch in the OFT, the ChR2 group mice explored the center area much less than mCherry control mice, and their time spent in the center area was significantly decreased (Figure 2G). Similarly, during the light‐on epoch in the EPM, the number of times into open arms and time spent in open arms in ChR2 group mice were significantly declined (Figure 2H). These data confirm that aBLA–vHPC glutamatergic projections bidirectionally modulated anxiety avoidance behaviors.

### 2.4 Activation of opCB1Rs at aBLA–vHPC Glutamatergic Terminals Reduces Anxiety Avoidance

**Figure 3:**
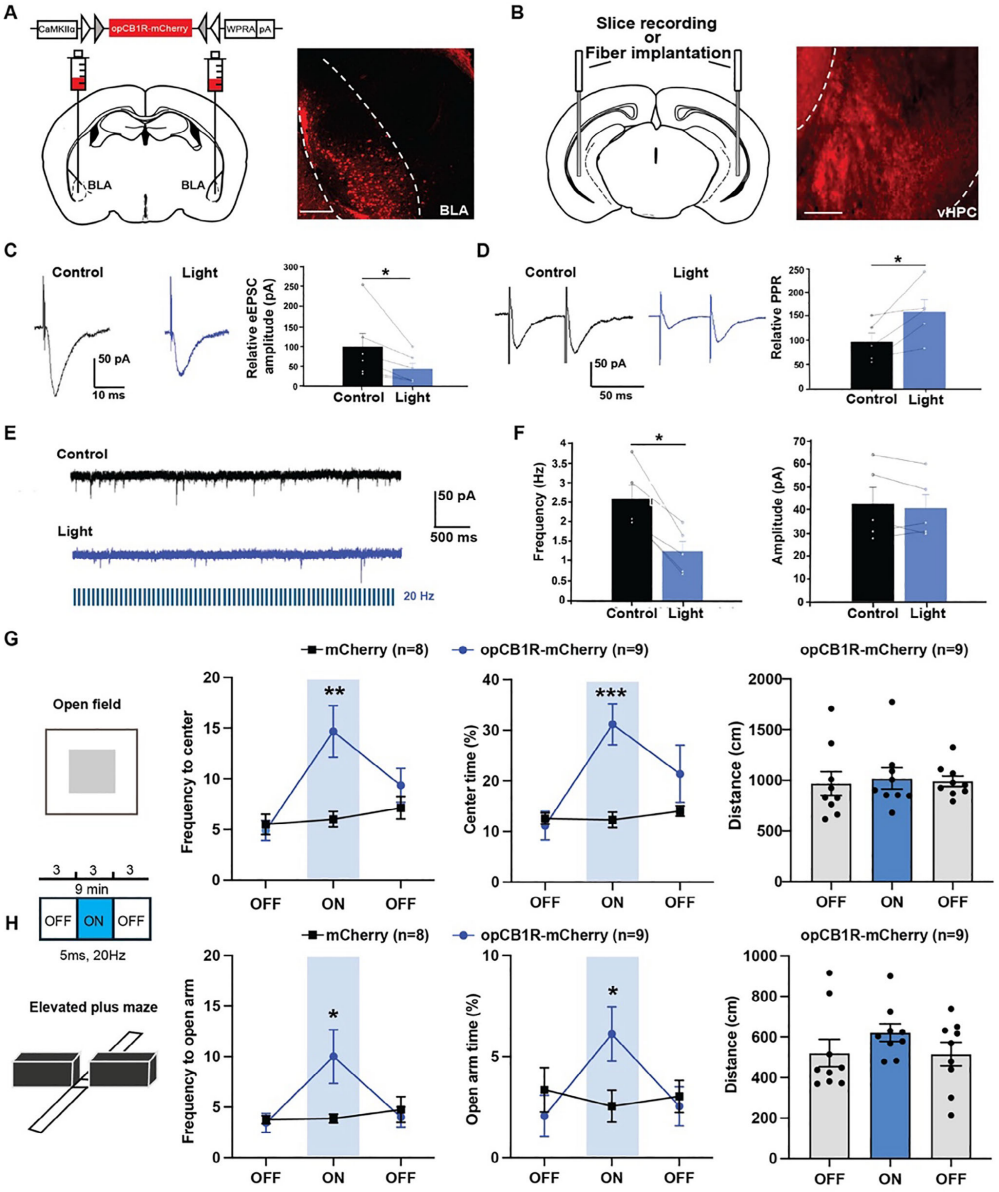
Specific activation of light‐sensitive opCB1Rs at anterior basolateral amygdala ventral hippocampus (aBLA–vHPC) glutamatergic terminals reduces anxiety avoidance. A) Left: Schematic diagram of CaMKIIα‐opCB1R virus injections into the aBLA. Right: Representative image showing expression of CaMKIIα‐ opCB1R virus in aBLA, scale bar = 100 µm. B) Left: Schematic diagram of recording optic fiber implantation into the vHPC. Right: Representative image showing expression of CaMKIIα‐ opCB1R virus in aBLA to vHPC terminals, scale bar = 100 µm. C,D) Representative raw traces of evoked excitatory post‐synaptic current (eEPSC) (C) and paired‐pulse stimulation‐evoked EPSC (D) were recorded before and after activation of opCB1R in aBLA–vHPC glutamatergic terminals. Relative eEPSC amplitude was reduced (C, paired *t*‐test, *n* = 5, ^*^ *p* < 0.05) and relative paired pulse ratio (PPR) was increased (D, paired *t*‐test, ^*^ *p* < 0.05). E) Representative raw traces of miniature excitatory postsynaptic current (mEPSC) were recorded before and after activation of opCB1R in aBLA–vHPC glutamatergic terminals. F) Quantification of mEPSC frequency (left) and amplitude (right), paired *t*‐test, *n* = 5, ^*^ *p* < 0.05. G) Quantification of frequency to center zone (2 × 3 mixed two‐way ANOVA, significant interaction between group and light on, F(2,30) = 8.550, *p* = 0.0012, ^**^ *p* < 0.01; independent *t*‐test, *t* = 3.071, df = 15, *p* = 0.0078, ^**^ *p* < 0.01), percentage time spent in the center zone of open field test (OFT) (2 × 3 mixed two‐way ANOVA, significant interaction between group and light on, F(2,30) = 9.862, *p* = 0.0005, ^***^ *p* < 0.001; independent *t*‐test, *t* = 4.138, df = 15, *p* = 0.0009, ^***^ *p* < 0.001) and total locomotor distance for opCB1R group. H) Quantification of frequency to open arm (2 × 3 mixed two‐way ANOVA, significant interaction between group and light on, F(2,30) = 5.393, *p* = 0.0100, ^**^ *p* < 0.01; independent *t*‐test, *t* = 2.143, df = 15, *p* = 0.0489, ^*^ *p* < 0.05), percentage time spent in the open arm of EPM (2 × 3 mixed two‐way ANOVA, significant interaction between group and light on, F(2,30) = 3.430, *p* = 0.0456, ^*^ *p* < 0.05; independent *t*‐test, *t* = 2.223, df = 15, *p* = 0.0420, ^*^ *p* < 0.05) and total locomotor distance for opCB1R group.

Because increased synaptic eCBs retrogradely activate presynaptic CB1Rs to suppress presynaptic neurotransmitter release,^[^ ^3^ ^]^ we hypothesized that the recently developed opCB1R strategy^[^ ^21^ ^]^ for a precise activation of CB1Rs in the circuit‐ and synapse‐specific manner is able to inhibit presynaptic release of glutamate at aBLA–vHPC circuits. The virus AAV‐CaMKIIα‐opCB1R‐mcherry was delivered into the aBLA, and 6 weeks later the vHPC slices were cut (**Figure** **3**A,B). To verify whether light stimulation can activate opCB1Rs, we recorded the electrically evoked excitatory post‐synaptic currents (eEPSCs). A concentrated stimulating electrode was placed 200–300 µm laterally to the recorded cell and electric stimulating pulses were given at 0.05 Hz. Picrotoxin (100 × 10^−6^ m) was included to block the effect of GABA_A_ receptors to ensure that only glutamate receptors were involved. At the same time, to activate opCB1Rs, postsynaptic recordings were made in areas of high opCB1R terminal infectivity in the vHPC with blue light (473 nm, 20 Hz). Light stimulation of opCB1Rs significantly reduced the amplitude of eEPSCs in postsynaptic neurons, indicating that light stimulation reduced the glutamate release (Figure 3C). To verify whether blue light was acting on the presynaptic receptor, we recorded the electrically evoked paired pulse ratio (PPR). Light stimulation of the opCB1R significantly increased the PPR, indicating a presynaptic effect (Figure 3D). To further confirm whether the effect of blue‐light stimulation of opCB1Rs mimics the activation of endogenous real CB1Rs, the CB1R agonist WIN55212 (10 × 10^−6^ m) was applied. Similarly, application of WIN55212 reduced the amplitude of eEPSCs (Figure S4A, Supporting Information) and simultaneously increased the PPR (Figure S4B, Supporting Information) in control mouse vHPC. These results suggest that after injection of AAV‐CaMKIIα‐opCB1R‐mcherry into aBLAs, opCB1Rs transported and expressed in aBLA–vHPC glutamatergic presynaptic terminals were activated by blue light stimulation to inhibit glutamate release. To further study the synaptic regulation of opCB1Rs in the aBLA–vHPC circuit, we recorded the miniature excitatory postsynaptic currents (mEPSCs). Light stimulation of opCB1Rs induced a decreased frequency of mEPSCs with unaffected amplitude (Figure 3E,F). Similar results were obtained after application of WIN55212 (Figure S4C, Supporting Information). Therefore, our results suggest that blue light stimulation of opCB1Rs at aBLA–vCA1 circuit terminals specifically reduced the presynaptic release of glutamate without significant effects on postsynaptic glutamate receptors.

While we and others showed that inhibition of aBLA–vHPC glutamatergic circuits suppressed anxiety avoidance, we hypothesized that activation of opCB1Rs at aBLA–vHPC terminals and subsequent decreased release of presynaptic glutamate would inhibit anxiety avoidance. To study this hypothesis, we delivered AAV‐CaMKIIα‐opCB1R‐mcherry into aBLAs and then implanted optic fibers into vHPCs (Figure 3A,B). Six weeks later, mice received optogenetic manipulation with 473 nm, 20 Hz 10 ms pulse. During the light‐on epoch, as compared with mCherry control mice, opCB1R mice explored the center area more often and spent more time in the center area in the OFT (Figure 3G). In the EPM, the frequency into open arms and time spent in open arms were also significantly increased in opCB1R mice (Figure 3H). These results suggest that activation of CB1Rs in aBLA–vHPC glutamatergic circuits robustly relieves anxiety avoidance

### 2.5 DAGLα Knockdown in aBLA–vHPC Circuits Aggravates Anxiety Avoidance

**Figure 4:**
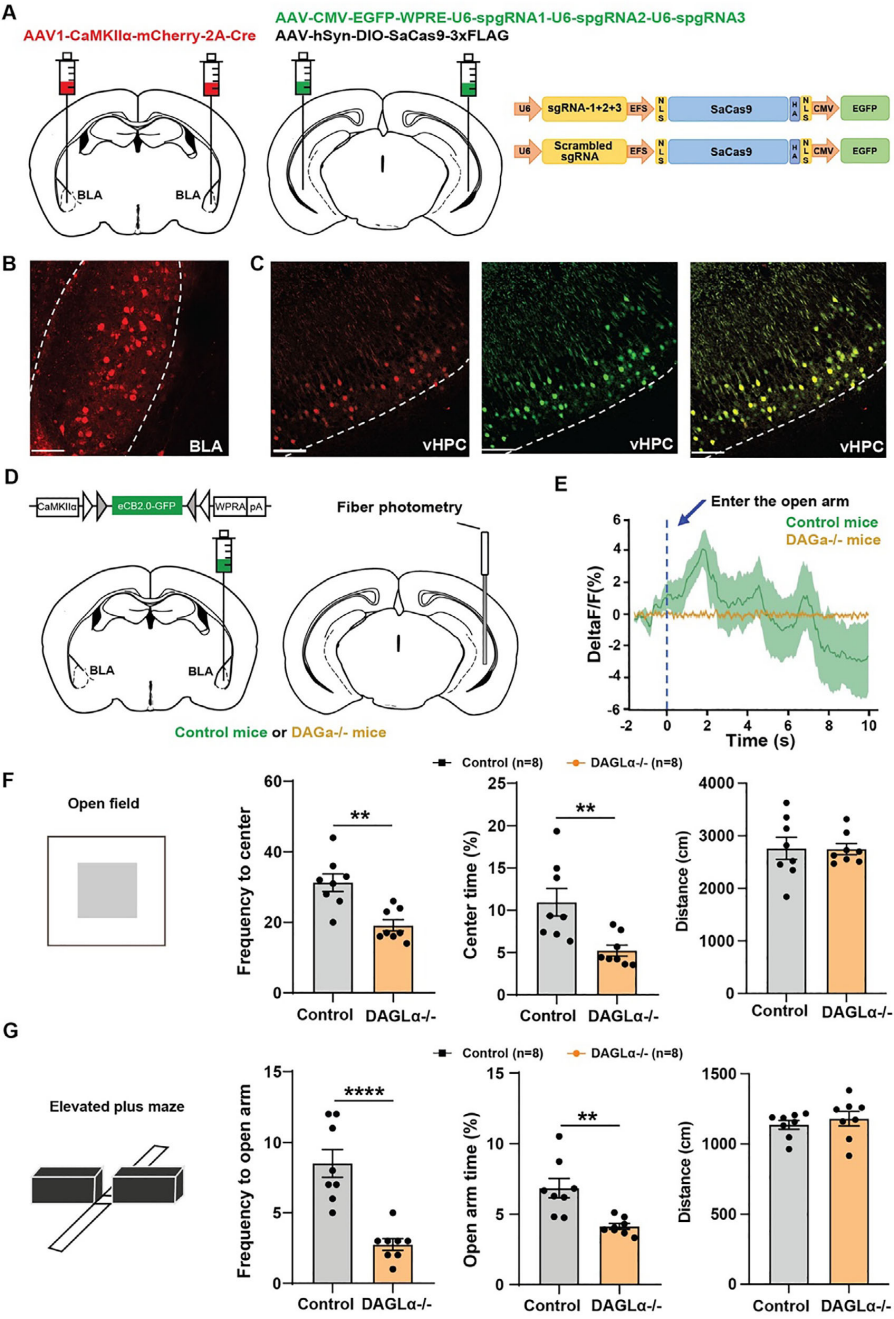
Specific knockdown of the diacylglycerol lipase α (DAGLα) in the anterior basolateral amygdal ventral hippocampus (aBLA–vHPC) glutamatergic circuit aggravates anxiety avoidance. A) Diagram of virus injections in aBLA and vHPC. B) Representative image showing the expression of AAV1‐CAMKIIα‐mcherry‐2A‐Cre in aBLA, scale bar = 100 µm. C) Representative image showing the expression of viruses in vHPC, scale bar = 100 µm. D) Left: Schematic diagram of CaMKIIα‐eCB2.0 virus injections into aBLA in control mice and aBLA–vHPC circuit‐specific DAGLα knockdown mice (DAGLα‐/‐). Right: Schematic diagram of recording optic fiber implantation into vHPC. Fiber photometry monitoring the endocannabinoids (eCB) release in aBLA–vHPC circuit was performed during elevated plus maze (EPM) test. E) Averaged trace for eCB release signals in control mice (green line) and DAGLα‐/‐ mice (yellow line). F) Quantification of frequency to center zone (independent *t*‐test, *t* = 4.081, df = 14, *p* = 0.0011, *n* = 8 for each group, ^**^ *p* < 0.01), percentage time spent in the center zone of open field test (OFT) (independent *t*‐test, *t* = 3.266, df = 14, *p* = 0.0056, *n* = 8 for each group, ^**^ *p* < 0.01) and total locomotor distance (independent *t*‐test, *t* = 0.6989, df = 14, *p* = 0.9453, *n* = 8 for each group). G) Quantification of frequency to open arm (independent *t*‐test, *t* = 5.400, df = 14, *p* < 0.0001, *n* = 8 for each group, ^****^ *p* < 0.0001), percentage time spent in the open arm of EPM (independent *t*‐test, *t* = 3.793, df = 14, *p* = 0.0020, *n* = 8 for each group, ^**^ *p* < 0.01) and total locomotor distance (independent *t*‐test, *t* = 0.7139, df = 14, *p* = 0.4870, *n* = 8 for each group). Data are shown as the mean ± SEM.

If light‐activated opCB1Rs in the aBLA–vHPC glutamatergic circuit can alleviate anxiety avoidance behaviors in mice, we hypothesized that reducing the 2‐AG level in this circuit could increase anxiety avoidance behaviors. To achieve this, we established a strategy to knockdown 2‐AG synthase DAGLα in a circuit‐specific manner. Thus, we expressed AAV1‐CaMKIIα‐mcherry‐2A‐Cre in aBLA glutamatergic neurons (**Figure** **4**A,B). Three weeks later, the mixed viruses of AAV‐CMV‐EGFP‐WPRE‐U6‐spgRNA1‐U6‐spgRNA2‐U6‐spgRNA3 and AAV‐hSyn‐DIO‐SaCas9‐3xFLAG were injected into bilateral vHPCs to specifically knockdown DAGLα in aBLA‐innervated vHPC neurons (Figure 4A,C). To verify that the eCB release is truly disrupted in DAGLα knockdown (DAGα‐/‐) mice, we used fiber photometry to monitor the eCB release in aBLA–vHPC glutamatergic circuits during the EPM test with CaMKIIα‐eCB2.0‐EGFP injection into bilateral aBLAs and optic fiber implantation into vHPCs (Figure 4D). When control mice were entering open arms, eCBs immediately released in aBLA–vHPC glutamatergic circuits (Figure 4E), which was similar to the results in Figure 1I. However, when entering open arms, DAGLα‐/‐ mice did not show the eCB release in aBLA–vHPC glutamatergic circuits (Figure 4E), suggesting the success of our DAGLα knockdown strategy. As compared to control mice, DAGLα‐/‐ mice showed a significant decrease of the frequency into and the time spent in the center area (Figure 4F). In the EPM test, as compared to control mice, DAGLα‐/‐ mice also showed a significant decrease of the frequency to and the time spent in open arms (Figure 4G). No significant changes of locomotor activities were detected in DAGLα‐/‐ mice in both OFT and EPM paradigms (Figure 4F,G). These data demonstrate that a specific reduction of 2‐AG levels in aBLA–vHPC glutamatergic circuits leads to increased anxiety avoidance. Additionally, we also investigated eCB effects in posterior BLA (pBLA) vHPC pathway. However, DAGLa knockdown in the pBLA‐vHPC pathway reduced anxiety avoidance, which was opposite to those in the aBLA–vHPC pathway. (Figure S5, Supporting Information). Therefore, eCB effects on anxiety avoidance in different subcircuits are different and even opposite, which enhance its circuit‐specific treatment.

### 2.6 Inhibition of aBLA‐Innervated vHPC Glutamatergic Neurons Alleviates Anxiety Avoidance

**Figure 5:**
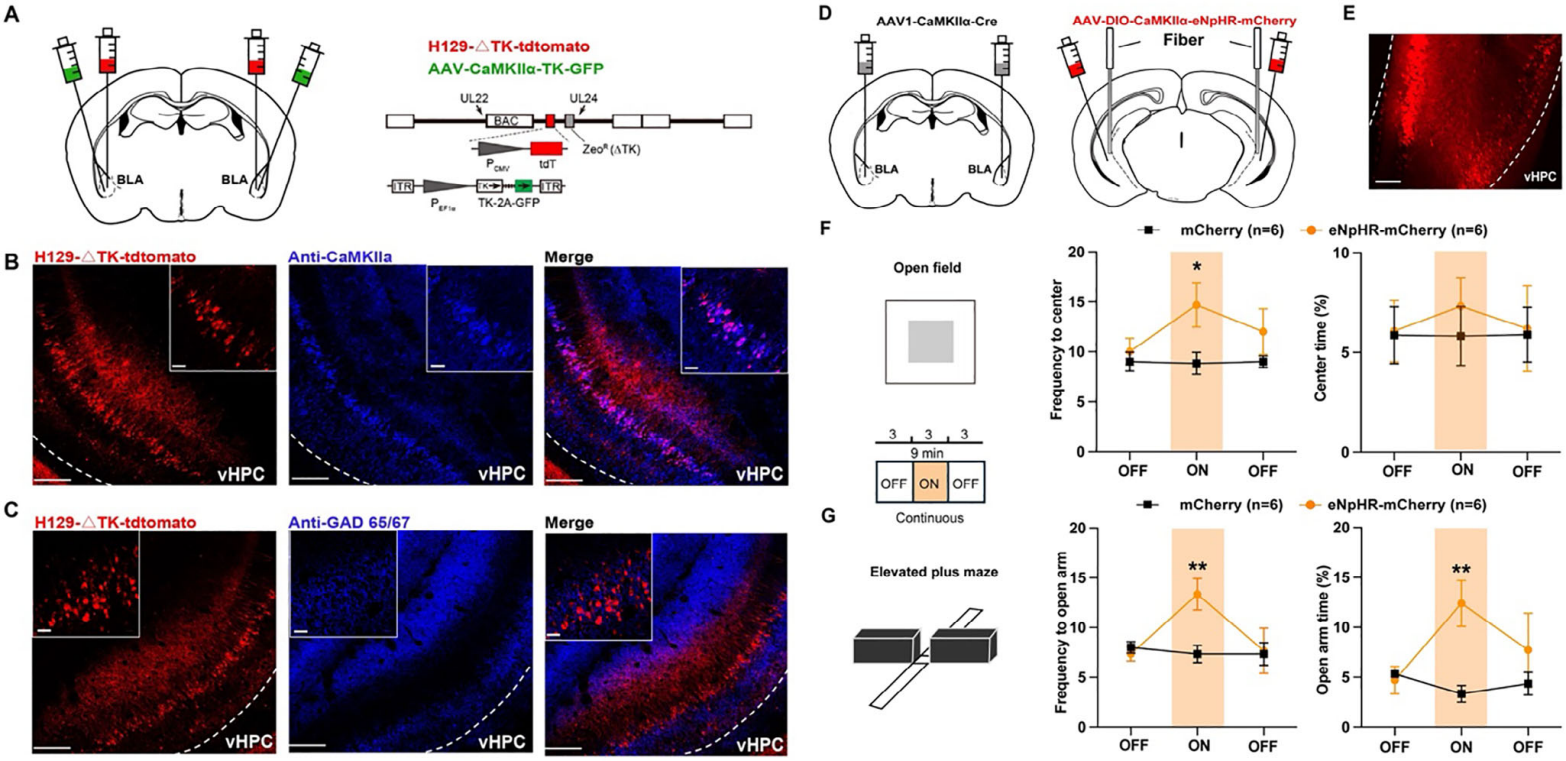
Selective inhibition of anterior basolateral amygdala (aBLA) innervated ventral hippocampus (vHPC) glutamatergic neurons alleviates anxiety avoidance. A) Diagram for anterograde trans‐monosynaptic virus injections into aBLA. B,C) TdTomato‐positive neurons in vHPC are aBLA‐innervated vHPC neurons, which are co‐immunostaining with the CaMKIIα (B) or GAD 65/67 (C) antibody, the makers of glutamatergic (B) or GABAergic (C) neurons in vHPC. Scale bar = 200 µm. Higher‐magnification images are provided in the top corner, Scale bar = 50 µm. D) Left: Diagram of AAV1‐CaMKIIα‐Cre virus injections into aBLA. Right: Diagram of DIO‐CaMKIIα‐eNpHR virus injections and optic fiber implantation in vHPC to selective inhibit aBLA‐innervated vHPC glutamatergic neurons. E) Representative image showing the expression of virus expression in vHPC, scale bar = 200 µm. F) Quantification of frequency to center zone (2 × 3 mixed two‐way ANOVA, no significant interaction between group and light on, F(2,20) = 1.895, *p* = 0.1764; independent *t*‐test, ON: *t* = 2.354, df = 10, *p* = 0.0403, ^*^ *p* < 0.05), percentage time spent in the center zone of open field test (OFT) (2 × 3 mixed two‐way ANOVA, no significant interaction between group and light on, F(2,20) = 0.4511, *p* = 0.6432; independent *t*‐test, ON: *t* = 0.7299, df = 10, *p* = 0.4822). G) Quantification of frequency to open arm (2 × 3 mixed two‐way ANOVA, significant interaction between group and light on, F(2,20) = 9.112, *p* = 0.0015, ^**^ *p* < 0.01; independent *t*‐test, ON: *t* = 3.308, df = 10, *p* = 0.0079, ^**^ *p* < 0.01), percentage time spent in the open arm of EPM (2 × 3 mixed two‐way ANOVA, no significant interaction between group and light on, F(2,20) = 3.240, *p* = 0.0604; independent *t*‐test, ON: *t* = 3.683, df = 10, *p* = 0.0042, ^**^ *p* < 0.01). Data are shown as the mean ± SEM.

To further identify neuron types in the vHPC innervated by aBLA glutamatergic neurons, we injected anterograde trans‐monosynaptic tracer H129‐△TK‐tdTomato into the aBLA 3 weeks after injection of the helper virus AAV‐CaMKIIα‐TK‐GFP into the BLA (**Figure** **5**A). Then, the mice were perfused 1 week later. aBLA‐innervated vHPC neurons were labeled as tdTomato‐positive neurons, which were largely overlapped with CaMKIIα antibody, but not GAD 65/67 antibody, indicating that aBLA‐innervated vHPC neurons are mainly glutamatergic neurons (Figure 5B,C).

Then, we examined the role of aBLA‐innervated vHPC glutamatergic neurons in anxiety avoidance behaviors. To achieve this aim, we injected the AAV1‐CaMKIIα‐Cre into bilateral aBLAs to make aBLA‐innervated vHPC neurons to express Cre recombinase. Then, we injected Cre‐dependent AAV2/9‐DIO‐CaMKIIα‐eNpHR3.0‐mCherry into bilateral vHPCs to specifically express the eNpHR in aBLA‐innervated vHPC glutamatergic neurons (Figure 5D,E). Three weeks later, approach and/or avoidance behaviors were recorded in the OFT and EPM with a light‐off/on/off 9‐min session. During the light‐on (596 nm, direct current) epoch in the OFT, we observed that as compared with mCherry group, the eNpHR3.0 group showed a significant increase of the number of times into and the time spent in the center area (Figure 5F). Similarly, during the light‐on epoch, eNpHR3.0 mice showed a significant increase of the number of times into and the time spent in open arms in the EPM (Figure 5G). These results demonstrate that specific inhibition of aBLA‐innervated vHPC glutamatergic neurons could decrease the anxiety avoidance.

## 3 Discussion

In recent years, the therapeutic potential of eCB signaling intervention in brain diseases has raised wide interest.^[^ ^24^ ^]^ The wide distribution of the eCB system in the amygdala and hippocampus, highlights its role in anxiety regulation. Thus, transcranial direct current stimulation produced anti‐anxiety responses in acute stress exposed rats via activation of amygdala CB1Rs^[^ ^25^ ^]^ and inhibiting the AEA degradation in the amygdala rapidly reduced anxiety.^[^ ^13^ ^]^ Early intervention with electroacupuncture prevented PTSD‐like behaviors in rats by enhancing eCB signaling in the hippocampus,^[^ ^26^ ^]^ while impairment of 2‐AG signaling in hippocampal glutamatergic neurons exacerbated anxiety‐like behaviors in mice.^[^ ^27^ ^]^ A recent study showed that administration of WOBE437, an inhibitor of 2‐AG and AEA reuptake, into the amygdala increased eCB levels at glutamatergic synapses to exert anxiolytic effect.^[^ ^28^ ^]^ Glutamatergic transmission in the amygdala correlates with anxiety states in mice, and 2‐AG‐mediated reductions in amygdala glutamatergic transmission are correlated with anxiolytic efficacy.^[^ ^29^ ^]^ Given that increasing eCB levels can alleviate anxiety, it raises the question of whether reducing circuit‐specific eCB levels would increase anxiety tendencies in mice. DAGLα is the enzyme responsible for synthesizing 2‐AG.^[^ ^30^ ^]^ The absence of DAGLα gene expression in cells prevents the production and secretion of 2‐AG.^[^ ^31^ ^]^ DAGLα knockout mice display varying degrees of anxiety and depressive tendencies.^[^ ^32^ ^]^ Inhibiting DAGLα also impairs the extinction of fear responses in mice.^[^ ^33^ ^]^ However, these studies used traditional pharmacological or gene knockout methods, lacking circuit‐ or synapse‐specificity, often leading to contradictory findings. For instance, some reports suggest that low concentrations of 2‐AG alleviate anxiety, while high concentrations tend to induce anxiety in both humans and rodents.^[^ ^9^, ^34^ ^]^ Systemic knockout of the 2‐AG hydrolase enzyme monoacylglycerol lipase congenitally increased 2‐AG levels in the amygdala, causing CB1R desensitization and compensatory damage to CB1R signaling, resulting in anxiety.^[^ ^35^, ^36^ ^]^ Given these complexities, a more detailed investigation of the role of eCB signaling in the BLA‐vHPC circuit in anxiety is necessary.

In the past decade, two studies showed an involvement of BLA‐vHPC glutamatergic circuits in anxiety.^[^ ^18^, ^19^ ^]^ We confirmed here that optogenetic inhibition and activation of aBLA–vHPC glutamatergic projections respectively reduced and increased anxiety avoidance, where the effect is robust and reversible in our OFF‐ON‐OFF paradigms. Using in vivo fiber photometry to monitor the real‐time neuronal activity changes, we further found that the activity of aBLA glutamatergic neurons and aBLA glutamatergic projections in the vHPC were immediately increased when mice were entering open arms of the EPM test. Interestingly, the duration of the activity increase in aBLA glutamatergic projections in the vHPC is significantly longer than that in aBLA glutamatergic neurons. The exact mechanism underlying such differences is unknown. The significantly increased activity of aBLA–vHPC glutamatergic circuits, particularly aBLA glutamatergic terminals in the vHPC, in response to open arm entering suggests a profound presynaptic glutamate release to activate postsynaptic neurons in the vHPC.

Because eCBs in the brain are activity‐dependently produced and released by postsynaptic neurons,^[^ ^3^ ^]^ activation of glutamatergic aBLA–vHPC postsynaptic neurons during anxiety avoidance may likely increase eCB release in aBLA–vHPC synapses. To address this hypothesis, we employed a recently developed probe, i.e., a genetically encoded eCB biosensor (GRABeCB2.0) consisting of a circular‐permutated EGFP and the human CB1Rs,^[^ ^20^ ^]^ which can monitor the real‐time dynamic eCB release in specific circuits and synapses. Based on advanced fluorescent protein technology, the design of this innovative tool is to capture the synaptic release of eCBs during neuronal activation with high sensitivity, thus giving us an unprecedented view of the dynamics of the eCB concentrations in synapses of living animals. We indeed observed that during the EPM test, eCBs were released in the aBLA–vHPC circuit immediately when mice were entering open arms. These findings were surprising, however, because increased synaptic release of eCBs can activate presynaptic CB1Rs to inhibit presynaptic release of glutamate in the aBLA–vHPC glutamatergic projection, leading to inhibition of this circuit and subsequent decrease of anxiety avoidance, while we showed increased circuit activity when mice entered open arms.

Nevertheless, the increased eCB release in aBLA–vHPC synapses is transient, whereas the increased activity of aBLA glutamatergic projections in the vHPC was long‐lasting. Therefore, the transiently reduced glutamate release in aBLA–vHPC synapses due to transient activation of presynaptic CB1Rs was only transiently counteracted profound and long‐lasting increase of glutamate release in aBLA–vHPC synapses when mice were entering open arms. These results suggest that the eCB signaling in this circuit serves as an intrinsic protective mechanism to transiently alleviate anxiety. If we profoundly activate eCB signaling in aBLA–vHPC circuits, the counteracting effect of eCB‐mediated intrinsic protective mechanism would overpass the naturally occurring increase of presynaptic glutamate release in aBLA–vHPC synapses, so as to reduce anxiety avoidance. To address this hypothesis, we employed opCB1R strategy that uses a light‐sensitive CB1R chimera by replacing the intracellular domains of bovine rhodopsin with those of human CB1Rs,^[^ ^21^ ^]^ resulting in a precise activation of opCB1Rs in the circuit‐ and synapse‐specific manner. The breakthrough of this technique is its ability to control the activation of CB1Rs in neuronal projections with millisecond accuracy, allowing us to simulate eCB signaling in aBLA–vHPC circuits for modulation of anxiety avoidance behavior. After administration of opCB1R virus into aBLAs and allowing it to transport to glutamatergic aBLA terminals in vHPCs, blue light activation of these opCB1Rs significantly inhibited glutamate release and reduced anxiety avoidance. Therefore, our results demonstrate that activation of eCB signaling in aBLA–vHPC circuits is indeed able to significantly decrease anxiety avoidance.

To provide further evidence supporting the contribution of eCB signaling in the aBLA–vHPC circuit to control anxiety avoidance, we tried to examine the effect of reducing eCB signaling in this circuit on anxiety avoidance. Thus, we utilized SaCas9 gene editing^[^ ^22^ ^]^ to knockdown the 2‐AG biosynthase DAGLα in a circuit‐ and synapse‐specific way.^[^ ^23^ ^]^ With this technique, we precisely reduced eCB synthase in aBLA–vHPC circuits to study the effect of decreased eCB levels on anxiety avoidance behavior. The high efficiency and specificity of SaCas9 make this process simple and reliable, providing a powerful tool for our research. As expected, using our new virus and strategy, we found that specific knockout of DAGLα in aBLA–vHPC circuits promoted anxiety avoidance. However, we also found that DAGLa knockdown in the pBLA‐vHPC pathway reduced anxiety avoidance, which was opposite to those in the aBLA–vHPC pathway. These results are consistent to prior literature.^[^ ^19^ ^]^ Therefore, eCB effects on anxiety avoidance in different subcircuits are different and even opposite, resulting in the global effects achieved by the preferable weight of anxiolytic versus anxiogenic.

The vHPC, a crucial node for controlling emotional and motivational behaviors,^[^ ^37^, ^38^ ^]^ primarily consists of glutamatergic neurons and GABAergic neurons.^[^ ^39^, ^40^, ^41^ ^]^ Using anterograde trans‐monosynaptic viral tracing^[^ ^42^ ^]^ combined with immunofluorescence staining, we revealed that approximately 80% of the aBLA‐innervated vHPC neurons are glutamatergic and specific inhibition of these aBLA‐innervated vHPC glutamatergic neurons significantly reduced anxiety avoidance.

## 4 Conclusion

In summary, a large number of studies have clearly indicated the important role of eCB signaling in the modulation of anxiety behaviors. However, the precise neural circuits of eCB modulation of anxiety remain unclear, which impedes the development of eCB‐based medicine. Moreover, the lack of techniques and tools for precise manipulation of eCB system in specific circuits and synapse also limited the related investigation. In this study, we employed three projection‐ and synapse‐specific eCB probes to monitor the real‐time dynamic eCB release, a precise activation of CB1Rs and a specific knockdown of 2‐AG biosynthesis enzymes in glutamatergic aBLA–vHPC circuits during anxiety avoidance. Thus, while inhibition and activation of aBLA–vHPC glutamatergic circuits respectively decreased and increased anxiety avoidance, these circuits showed eCB release during anxiety avoidance, activation of CB1Rs at these circuits reduced anxiety avoidance, and knockdown of eCB biosynthesis at these circuits increased anxiety avoidance. These findings establish a base for future clinical interventions of anxiety disorders with precise interference of eCB signaling in aBLA–vHPC circuits.

## 5 Experimental Section

### Animals

Adult C57BL/6 J male mice (8–16 weeks old) were purchased from Beijing Vital River Laboratory Animal Technology Co., Ltd. Before surgery, these mice were socially housed in groups of five per cage, which were exposed to a 12‐h light‐dark cycle (light hours: 7:00 a.m.–19:00 p.m. at a stable temperature 23–25 °C) and fed and watered ad libitum. After surgery, mice were singly housed. All behavioral tests were conducted during the period of light and kept in a quiet environment. The animal experimental procedures in this study strictly followed Qingdao University animal ethical requirements.

### Viruses

For fiber photometry recordings. AAV2/9‐CaMKIIα‐GCaMP6(s)‐WPRE‐pA (viral: 3.64 × 10^12^ particles mL^−1^) were purchased from Obio Technology (Shanghai) Corp., Ltd. AAV9‐CaMKIIα‐eCB 2.0‐GFP (viral: 1.09 × 10^13^ particles L^−1^) were purchased from VigeneBio (Jinan).

For circuit‐specific eCB system manipulation experiments. AAV2/9‐CaMKIIα‐opCB1R‐mCherry (viral: 7.43 × 10^12^ particles mL^−1^) were constructed by Jiangfan Chen's lab at Wenzhou Medical University, China. The optoCB1R was developed by replacing the intracellular the loops 1, 2, and 3 of bovine rhodopsin with those from human CB1R based on established optoXR strategies. The exact sequence of optoCB1R can be found in the paper published by Jiangfan Chen's lab.^[^ ^21^ ^]^ AAV2/9‐hSyn‐DIO‐SaCas9‐3xFLAG (viral: 4.32 × 10^13^ particles mL^−1^) and AAV2/9‐CMV‐EGFP‐WPRE‐U6‐spgRNA1‐U6‐spgRNA2‐U6‐spgRNA3 (viral: 7.23 × 10^12^ particles mL^−1^) were custom synthesized by Obio Technology (Shanghai) Corp., Ltd, and mixed at 1:1 ratio for injection.

For optogenetics experiment. AAV2/9‐CaMKIIα‐ChR2‐mCherry (viral: 1.56 × 10^12^ particles mL^−1^), AAV2/9‐CaMKIIα‐eNpHR3.0‐EGFP (viral: 4.45 × 10^13^ particles mL^−1^), DIO‐CaMKIIα‐eNpHR‐mCherry (viral: 1.02 × 10^12^ particles mL^−1^) and AAV2/1‐CaMKIIα‐mCherry‐2A‐Cre (viral: 4.38 × 10^12^ particles mL^−1^) were purchased from Obio Technology (Shanghai) Corp., Ltd.

Anterograde trans‐monosynaptic tracing viruses. AAV2/9‐CaMKIIα‐TK‐GFP (viral: 2.0 × 10^12^ particles mL^−1^) and H129‐△TK‐tdTomato (viral: 1.4 × 10^9^ particles mL^−1^) were purchased from BrainVTA, Wuhan.

All viruses are stored at −80 °C, and repeated freezing and thawing is strictly prohibited.

### Viral Injection, Stereotaxic Surgery, and Optical Fiber/Cannula Implantation

Mice were deeply anesthetized and placed in a stereotaxic apparatus (RWD, Shenzhen, China). During surgery and virus injection, isoflurane (1%) was used to maintain anesthesia. A dental drill was used to thin the skull above the target area, creating a small hole to expose the brain. Injections were performed with a 10 mL syringe (Hamilton, Nevada, USA) connected to a glass micropipette with a 10–15 mm diameter tip. Syringe pumps (KD Scientific, 78–8130, USA) were used to inject the viruses for speed and volume control. The coordinates were measured from the bregma according to the mouse atlas.

For bilateral stereotaxic injection of viruses into the aBLA (AP: −1.3 mm; ML: ±3.25 mm; DV: −4.5 mm relative to bregma; AP, ML, and DV denote anteroposterior, mediolateral, and dorsoventral distance from the bregma, respectively) or vHPC (AP: −3.16 mm; ML: ±3.00 mm; DV: −4.0 mm relative to bregma), 100 nL of virus was injected into each location at a rate of 60 nL min^−1^. The syringe should be remained in place for 10 min following the completion of injection to wait the dissemination of viruses and then gradually removed. Approximately 3 weeks later, mice were prepared for optical fiber or cannula implantation.

For optical fiber/cannula implantation, optical fibers or cannulas (RWD Life Sciences) were unilaterally or bilaterally implanted above the BLA or vHPC. Two stainless steel screws were implanted in the skull for support. The optical fiber/cannulas and screws were firmly secured using dental cement to ensure stability. Stainless steel occluders were inserted into each cannula to prevent any potential blockages. After surgery, the mice were kept on a heating pad at 37 °C until they recovered. After the animals were awake, they were singly housed and relevant behavioral experiments were performed after 1 week. Only mice with the correct locations of optical fibers/cannulas and viral expression were used for further analysis.

### In Vivo Fiber Photometry Recording

For calcium signal recording, the activities of aBLA glutamatergic neurons and aBLA–vHPC glutamatergic projections during anxiety behavior test were measured using fiber photometry, with AAV2/9‐CaMKIIα‐GCaMP6(s)‐WPRE‐pA virus injected and optic fiber implanted.

For eCB release recording of aBLA–vHPC glutamatergic circuit, after 4 weeks expression of AAV–CaMKIIα ‐eCB 2.0‐GFP, optic fibers (Diameter 200 µm, Numerical aperture 0.37 mm, FOC‐C‐1.25‐200‐0.37‐6.0, Inper, China) were placed into the vHPC and used to record the release of eCB in mice during anxiety behavior test.

The fiber photometry recording system's light source emitted excitation light at 473 nm (Thinker Tech Nanjing Bioscience Inc), which was reflected off a dichroic mirror and focused into a multimode optical fiber by an objective lens. This fiber conveyed the excitation light to targeted brain areas in the mice. To prevent photobleaching, the laser intensity was maintained within the range of 0.01 to 0.03 mW at the fiber tip‐to‐animal tissue interface. When calcium or eCB is released from aBLA glutamatergic neurons or aBLA–vHPC glutamatergic terminals specifically labeled by the calcium indicator GCaMP6s or eCB 2.0 sensor, the fluorescent signal becomes stronger and is collected by a photomultiplier after high‐pass filtering. The current generated by the photomultiplier was then converted into a voltage signal by an amplifier, which could be captured and further processed through a low‐pass filter with a cutoff frequency below 100 Hz. The captured data was visualized and analyzed using MATLAB. To express the change in fluorescence intensity before and after an event, the ratio of the difference between the current fluorescence intensity (*F*) and the baseline fluorescence intensity (F0) to the baseline fluorescence intensity (F0), namely, Δ*F*/F0 = (F − F0)/F0, was calculated. The event‐related line graph showed the average results of multiple experiments.

### Ex Vivo Electrophysiology Recording

Mice (8–10 weeks old) were deeply anesthetized with isoflurane (1.5–3.0%) and perfused with ice‐cold oxygenated (95% O_2_ and 5% CO_2_) cutting artificial cerebrospinal fluid (ACSF) consisting of (in × 10^−3^ m): 93 NMDG, 93 HCl, 2.5 KCl, 10 MgSO_4_ · 7H_2_O, 1.2 NaH_2_PO_4_, 30 NaHCO_3_, 25 D (+)Glucose, 20 HEPES, 5 Na ascorbate, 3 Na pyruvate, 2 Thiourea and 1 CaCl_2_ (pH 7.4). Brains were removed quickly, and 250 mm thick coronal slices of the vHPC were prepared with a vibratome (Leica, VT1200, Germany) in cutting ACSF. The slices were recovered for 60 min at 36 °C (TC‐324B; Warner) in oxygenated incubating ACSF containing (in × 10^−3^ m): 126 NaCl, 2.5 KCl,1.25 NaH_2_PO_4_ ·2 H_2_O, 25 NaHCO_3_, 2 CaCl_2_ ·2H_2_O, 2 MgSO_4_ ·7 H_2_O, 10 D (+)‐Glucose, and then maintained at room temperature with 95% O_2_/5% CO_2_ gas. Whole‐cell patch‐clamp recordings were performed at room temperature with a MultiClamp 700B amplifier (2 kHz low‐pass filtered, 10 kHz digitization, Molecular Devices, USA) and a 1440A interface (Molecular Devices, USA) with pClamp 10.4 software (Molecular Devices, USA). Fluorescent cells were visualized under a Nikon Eclipse FN1 microscope (Japan) equipped with a 40× water‐immersion lens and illuminated with a mercury lamp. Whole‐cell patch‐clamp recordings were used unless otherwise stated. Data were collected 2 min after obtaining a stable whole cell configuration.

To verify the feasibility of opCB1R virus in aBLA–vHPC glutamatergic terminals, postsynaptic recordings in voltage‐clamp mode (holding potential, 70 mV) were made in the vHPC. The opCB1R virus can be activated with 473 nm blue light emitted by an LED (Polygon400, Mightex) through the objective lens at a light intensity < 20 mW. Electrodes (3–5 MU) was filled with an internal solution containing (in × 10^−3^ m): 110 potassium gluconate, 133 K‐gluconate, 18 NaCl, 10 HEPES, 2 Mg‐ATP, 0.3 Na_3_‐GTP, and 0.6 EGTA. An electrical stimulation electrode was placed near the vHPC at a distance of 0.2–0.3 mm from the recorded neurons under an infrared visual phase contrast microscope. Subsequently, a 0.05 Hz stimulus pulse was applied, and the stimulus intensity was fine‐tuned to elicit an excitatory postsynaptic current (EPSC) amounting to 30%–40% of the maximal amplitude. To observe the PPR, pairs of stimuli were applied at a frequency of 0.05 Hz with brief inter‐stimulus intervals (50 ms) to elicit two synaptic responses. Moreover, mini‐EPSCs were recorded in the presence of tetrodotoxin (TTX, 1 × 10^−6^ m, MedChemExpress) and picrotoxin (100 × 10^−6^ m). All action potential properties and excitability recordings were performed in the presence of 100 × 10^−6^ m picrotoxin.

### Open‐Field Test (OFT)

The open field box (40 × 40 × 40 cm, length × width × height) was made of transparent plastic and a 20 × 20 cm square was positioned at the center to serve as the central zone, with the remaining area of the box designated as the peripheral zone. Individual mice were placed in the center of the box before starting the session, and the trajectories were captured using a video camera and meticulously analyzed employing Noldus software. In the optogenetic experiments, mice were tested in a single 9‐min session with three 3‐min epochs. The test began with a light‐off baseline epoch, followed by a light‐on illumination epoch, and concluded with a second OFF epoch. During the light‐on epoch, a constant yellow light (10 mW, 589 nm) or 20 Hz blue light (10 ms pulses, 10 mW, 473 nm) was delivered to vHPC through the optical fibers. For DAGLα knockout experiments, mice were permitted to explore freely for 6 min. The frequency of mice entering the central area, the percentage of time spent in the central area, and the total distance moved were analyzed. After each experiment, the box was cleaned with 75% ethanol to remove the previous mouse's odor, and the next mouse started after the alcohol had evaporated.

### Elevated Plus Maze (EPM)

EPM is a cross‐shaped maze device, consisting of two open arms (dimensions: 30 cm × 10 cm) and two closed arms (dimensions: 30 × 10 × 10 cm) intersecting at 90° in the form of a plus, and elevated 50 cm from the floor. Individual mice were positioned at the center cross (10 × 10 cm^2^) facing one of the two open arms before starting the session. In the optogenetic experiment, the 9‐min session was the same as that in OFT. For DAGLα knockout experiments, mice were permitted to explore freely for 6 min. Their movement trajectories were captured with a video camera and analyzed using Noldus software. Statistical analysis was conducted on the frequency of mice entering and exiting the open arm, the percentage of time spent in the two open arms, and the total locomotor distance. In the fiber photometry recording experiment, the fluorescence signals were recorded continuously for 6 min, and the changes of fluorescence signals were analyzed when the mice entries into the open arms. After each experiment, the box was cleaned with 75% ethanol to remove the previous mouse's odor, and the next mouse started after the alcohol had evaporated.

### Reagents and Antibodies

WIN55212(CB1 receptor agonist, APExBIO) and AM281 (CB1 receptor antagonist, APExBIO) were centrifuged and dissolved to a concentration of 0.5 µg/0.1 µL with 0.9% saline, Tween 80, and dimethyl sulfoxide (DMSO) at a ratio of 9:0.5:0.5, and stored at −20 °C. Picrotoxin (GABA receptor inhibitor, APExBIO) was dissolved in DMSO to make a 100 × 10^−3^ m masterbatch, diluted to 100 × 10^−6^ m for experiments, and stored at −20 °C. TTX (Tocris), dissolved in DMSO to make a masterbatch of 1 × 10^−3^ m, the final concentration used in the experiments was 0.5 µg µL^−1^, and stored at −20 °C. Primary antibody: CaMKIIα antibody (1:250) and GAD65/67 antibody (1:100) both were purchased from Abcam, stored at −20 °C. Secondary antibody: HRP‐labeled donkey anti‐rabbit IgG (1:500) were purchased from Thermo Fisher Scientific, stored at 4 °C.

### Immunohistochemistry

Animals that had undergone behavioral analysis were anesthetized and perfused with 0.9% saline and 4% paraformaldehyde. Brains were carefully extracted and immersed in 4% paraformaldehyde for overnight fixation. Following this, they were dehydrated in a 30% sucrose solution at 4 °C for 48 h. Utilizing a cryostat microtome, the brains were sectioned to a precise thickness of 30 µm. Slices selected for viral fluorescence observation were rinsed three times with PBS, and then directly mounted using a sealing solution enriched with DAPI. These preparations were subsequently examined and imaged on Olympus VS120 (Japan) virtual slice microscope system or Nikon A1 confocal microscope (Japan) with a laser confocal microscope (OLYPUS, Japan) for detailed analysis. Evaluation of the optic fibers’ position was based on the lesions in the tissue created by the fiber tip.

Slices selected for immunohistochemistry staining were rinsed three times with PBS, with each wash lasting 10 min. Subsequently, PBST containing 0.1% Triton was prepared by incorporating Triton into PBS. The sections were then incubated with 5% donkey serum (dissolved in PBST) at room temperature for 2 h to block non‐specific binding. Primary antibodies (anti‐CaMKIIα 1:250, anti‐GAD65/67 1:100) were added and incubated overnight at 4 °C on a shaking bed. After a 30‐min recovery of the primary antibody at room temperature, it was washed three times with PBS on a shaker for 10 min each time. Subsequently, HRP‐labeled donkey anti‐rabbit IgG fluorescent secondary antibody (1:500, Thermo Fisher) and Alexa Fluor 640 (711‐545‐152/712‐005153, Molecular Probes) were added. The samples were incubated at room temperature for 2 h in the dark, followed by three washes with PBS on a shaker for 10 min each. After mounting with a DAPI‐containing sealing solution, the fluorescence staining was observed using a laser confocal microscope.

### Statistical Analyses

The commercial software GraphPad Prism version 9 was used for statistical comparisons. Group differences were detected using either one‐way ANOVA with Tukey's post hoc tests or mixed two‐way repeated‐measures ANOVA. Independent and paired two‐sample Student's *t*‐test were used for two‐group comparisons. Data are presented as means ±​ S.E.M. Statistical significance was set at ^*^ *p* <​ 0.05, ^**^ *p* <​ 0.01, ^***^ *p* <​ 0.001. The statistical analysis methods were indicated in figure legends. All experiments and data analyses were conducted blindly. The size of the animal sample was determined by the estimated variance of the experiment and previous experience in similar studies. Random method was used to assign experimental groups. The number of experimental replicates (*n*) is indicated in the figures and refers to the number of experimental subjects independently treated in each experimental condition.

## Conflict of Interest

The authors declare no conflict of interest.

## Author Contributions

B.X. and M.‐X.Z. contributed equally to this work, and they are co‐first authors. B.X., M.‐X.Z., X.‐C.B., S.‐P.L., X.‐T.B., and F.G. performed the research; B.X., M.‐X.Z., Y.D., Y.W., J.‐F.L., and F.G. worked on the data analysis and validation; X.Z. and Y.W. conceived the project and wrote the manuscript.

## Supporting information



Supporting Information

## Data Availability

The data that support the findings of this study are available from the corresponding author upon reasonable request.
