# Supplementary material for: An Amygdala‐hippocampus Circuit for Endocannabinoid Modulation of Anxiety Avoidance
Source: Adv Sci (Weinh). 2025 Jun 16;12(34):e05121. doi: 10.1002/advs.202505121 (PMC12442602; doi:10.1002/advs.202505121)
Supplement: Supplementary file 1 — Supporting Information [file ADVS-12-e05121-s001.docx]

Supporting Information

An amygdala-hippocampus circuit for endocannabinoid modulation of anxiety avoidance

Bao Xue^#^, Mao-Xing Zhang^#^, Xiao-Chen Bi, Shou-Peng Lai, Xin-Tian Bie, Yuan Dong, Jian-Feng Li, Fang Gao, Xia Zhang*, Ying Wang*


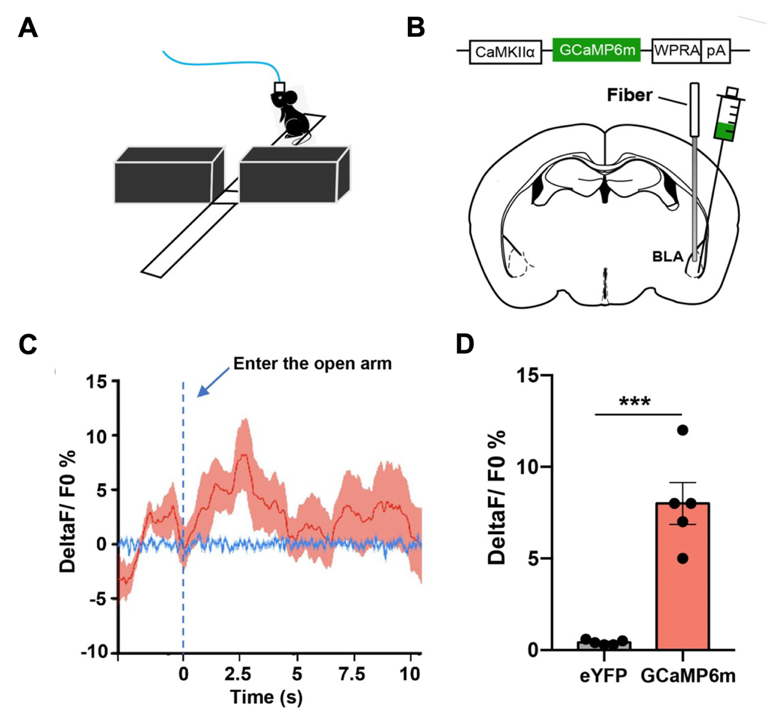


**Figure S1.** The activity of BLA glutamatergic neurons is stress responsive. (A) Diagram of fiber photometry recording in EPM test. (B) Schematic diagram of CaMKIIα-GCaMP6m virus injections and recording optic fiber implantation into the BLA. (C) Averaged trace for calcium signals in GCaMP6m mice (red line) and eYFP mice (blue line). The vertical dashed line indicates the mice entering the open arm. (D) Quantification of open arm stress-induced activation peak deltaF/F (%) in GCaMP6m mice and eYFP mice. Students t-test, ***P<0.001.


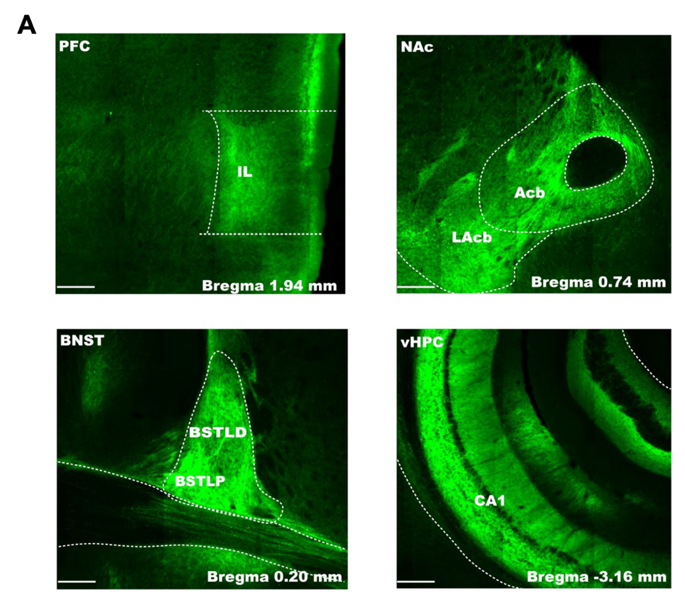


**Figure S2.** The outputs of BLA glutamatergic neurons in downstream areas. (A) BLA glutamatergic neurons send projections to PFC, NAc, BNST, vHPC. PFC: prefrontal cortex, NAc: nucleus accumbens, BNST: bed nucleus of the stria terminalis, vHPC: ventral hippocampus.


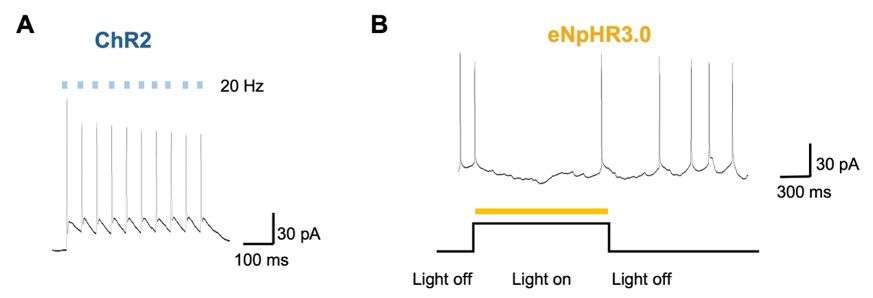


**Figure S3.** Functional validation for ChR2 and eNpHR3.0. (A) In *in vitro* electrophysiological study, 20 Hz blue light stimulation could activate ChR2-positive BLA glutamatergic neurons. (B) In *in vitro* electrophysiological study, direct yellow light stimulation could inhibit eNpHR3.0-positive BLA glutamatergic neurons.


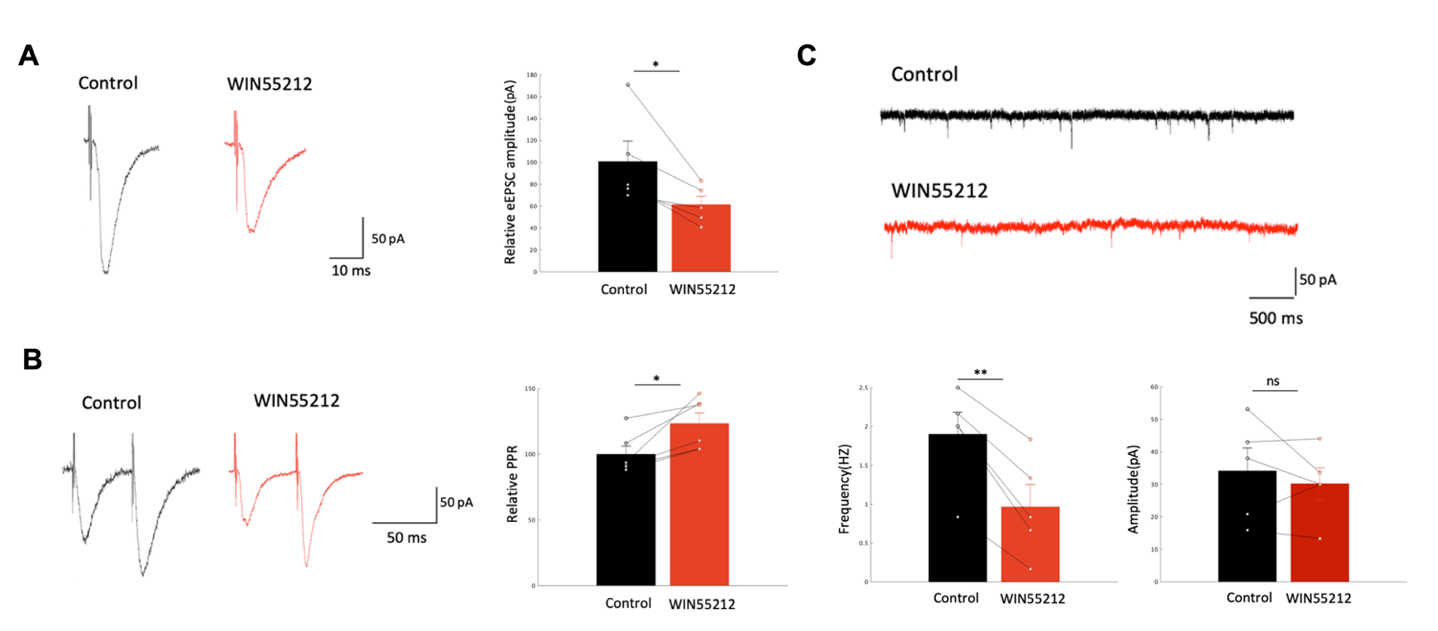


**Figure S4.** CB1 receptor agonist WIN55212 could reduce glutamate release in vHPC. (A-B) Representative raw traces of eEPSC (A) and paired-pulse stimulation-evoked EPSC (B) were recorded before and after application of WIN55212 (100nM) in vHPC brain slice. Relative eEPSC amplitude was reduced (A, paired *t*-test, *P<0.05) and relative PPR was increased (B, paired *t*-test, *P<0.05). (C) Top: representative raw traces of mEPSC were recorded before and after application of WIN55212 (100nM) in vHPC brain slice. Bottom: quantification of mEPSC frequency (left) and amplitude (right), paired *t*-test, **P<0.01.


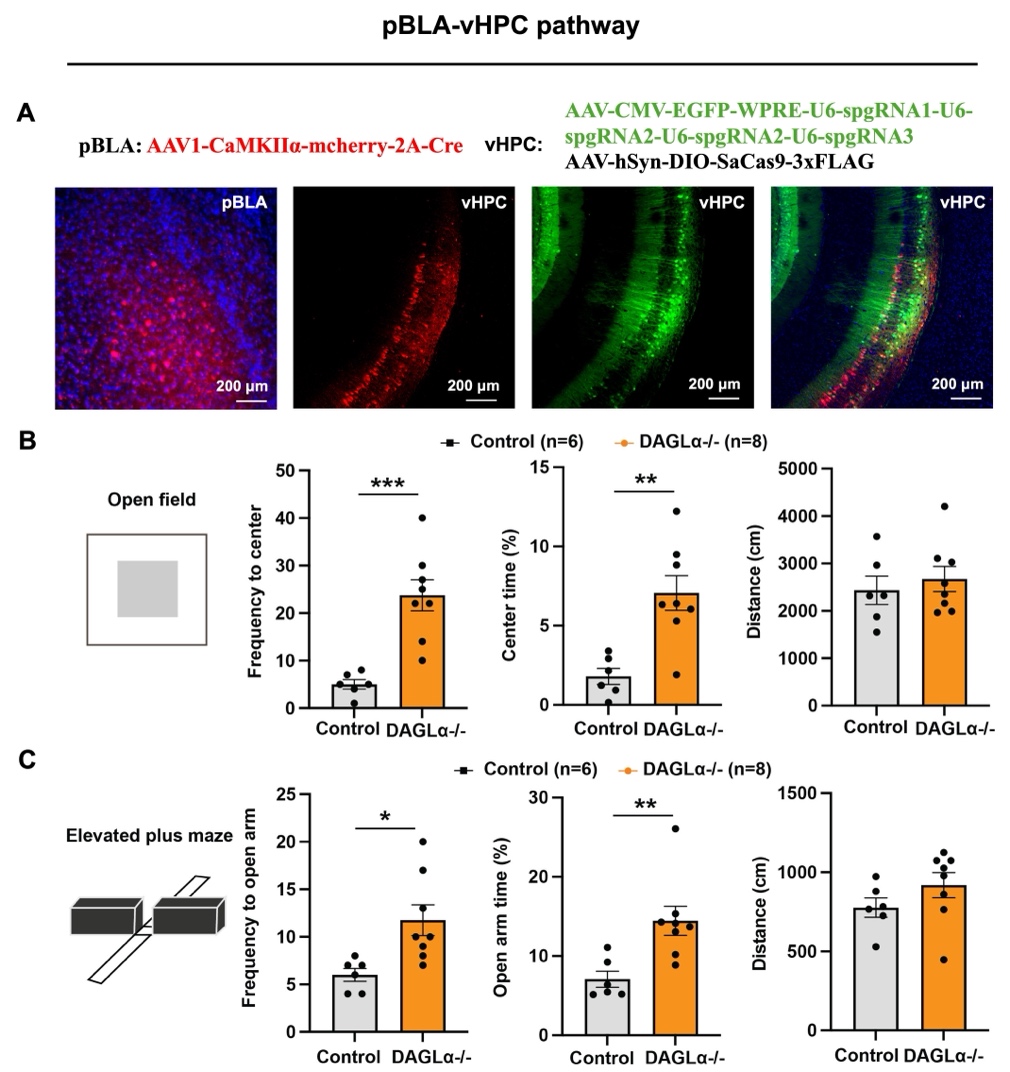


**Figure S5.** Specific knock down of the DAGLα in the pBLA-vHPC glutamatergic circuit reduces anxiety avoidance. (A) Representative image showing the expression of AAV1-CAMKIIα-mcherry-2A-Cre in pBLA and the combined expression of viruses in vHPC, scale bar = 200 μm. (B) Quantification of frequency to center zone (independent t-test, t=4.771, df=12, P=0.0005, ***P<0.001), percentage time spent in the center zone of OFT (independent t-test, t=3.896, df=12, P=0.0021, **P<0.01) and total locomotor distance (independent t-test, t=0.5925, df=12, P=0.5645). (C) Quantification of frequency to open arm (independent t-test, t=2.902, df=12, P=0.0133, *P<0.05), percentage time spent in the open arm of EPM (independent t-test, t=3.199, df=12, P=0.0076, **P<0.01) and total locomotor distance (independent t-test, t=1.335, df=12, P=0.2066). Data are shown as the mean ± SEM.
